# Investigation of the effect of task-oriented occupational therapy on daily living activity performance in chronic stroke patients

**DOI:** 10.12669/pjms.40.6.7954

**Published:** 2024-07

**Authors:** Cigdem Cekmece, Ilgin Sade, Elif Ozcan, Sibel Balci

**Affiliations:** 1 Cigdem Cekmece, PhD. Associate Professor, Section of Occupational Therapy, Department of Therapy and Rehabilitation, Vocational School of Kocaeli Health Services. Instute of Health Sciences of Kocaeli University, Kocaeli, Turkey; 2 Ilgin Sade, MD. Associate Professor, Department of Physical, Medicine and Rehabilitation, Faculty of Medicine, Instute of Health Sciences of Kocaeli University, Kocaeli, Turkey; 3 Elif Ozcan PT & Master degree Student Instute of Health Sciences of Kocaeli University, Kocaeli, Turkey; 4 Sibel Balci Associate Professor, Biostatistics and Medical Informatics, Faculty of Medicine Instute of Health Sciences of Kocaeli University, Kocaeli, Turkey

**Keywords:** Occupational therapy, Upper extremity, Stroke, Task-oriented therapy

## Abstract

**Objective:**

Task-oriented therapy (TOT) is used to increase the effectiveness of upper extremity (UE) in activity daily living (ADL). This study aimed to investigate the effect of TOT on the participation and ADLs of stroke patients.

**Methods:**

Between October 2018 and February 2019, 28 chronic stroke patients were included in the study treated in Kocaeli University Hospital, Department of Physical Medicine and Rehabilitation, Turkey. The performance areas and participation status of the patients in which they experienced limitations were evaluated with the Canadian Occupational Performance Scale (COPM), and their level of independence in ADLs was evaluated with the Modified Frenchay Activity Index (mFAI) and Barthel Index (BI). All patients were included in the occupational therapy (OT) program five days a week for three weeks at Kocaeli University Hospital. Three ADLs in which they had difficulties were studied with 28 patients. Each of the activities was designed specifically for the patient. All assessments were repeated after three weeks of treatment.

**Results:**

A total of 28 patients, 12 females and 16 males, diagnosed with stroke, were included in the study. A statistically significant increase was found in the COPM performance and satisfaction value compared to the pre-treatment value (p<0.001). A statistically significant difference was found between pre- and post-treatment mFAI and BI values (p<0.001).

**Conclusions:**

Adding task-oriented therapy to the rehabilitation programs of stroke patients will contribute to the improvement of ADL.

## INTRODUCTION

Stroke, a significant cause of disability among adult population, has become an important public health problem with its increasing incidence.^1^ Permanent physical and social functional loss develops in at least half of stroke patients

It has been reported that the UE is affected in 80% of patients in the acute period, and 50-75% of the UE limitation continues in the sixth month after stroke. It is stated that UE functions are fully restored in only 5% of the patients.^2^ These limitations in the UE seriously affect the participation of patients in task-oriented activities in daily life.^3^ Contractures and deformities that may occur in patients can be reduced, muscle strength can be increased, and ambulation ability can be improved with classical conventional treatments. However, in many important activities such as dressing, eating, and bathing in daily life, the patient may continue to face difficulties.^3^

Functional recovery after stroke is associated with the brain’s neuroplasticity capacity. In recent years, it has been shown that functional training of the plegic side applied in the acute and subacute periods contributes to the development of neuroplasticity.^4^ In order to improve the physical and emotional functions, quality of life and social participation levels of individuals with stroke, it is necessary to define the limitations created by the disease very well. As a result of the evaluations, it is also important to determine priorities within the framework of ICF (International Classification of Functioning, Disability and Health) and to determine near-term and long-term goals while creating the rehabilitation plan. In most of the studies conducted in recent years, it is emphasized that it is not enough to treat patients only with rehabilitation applications for physical problems and symptoms, but it is also important for individuals to take part in life by increasing their level of independence in daily life.^5^

Another method used to increase functionality in stroke is task-oriented therapy (TOT). TOT of the UE includes patient-specific, mostly bilateral occupational therapy studies that encourage the functional use of the plegic side in line with the needs of stroke patients in their daily living activities. In recent years, TOT has begun to be used to increase functionality in neurological diseases such as stroke^6^, Multiple Sclerosis^7^ and Parkinson’s disease.^8^ TOT is a treatment model that includes different motor tasks related to daily living activities, such as maintaining balance, walking, and UE reaching functions. Researchers state that these tasks that make up TOT are more intense and specific than traditional physiotherapy, allowing the tasks to be practiced repeatedly to achieve and maintain motor learning.^9^,^10^ In the study conducted by Annino G et al. and colleagues, it was stated that TOT can increase the excitability of the motor area in the brain, which can positively contribute to the increase of motor control.^11^ Studies in the literature show that TOT contributes to neuroplasticity and increases functional development in the UE. However, it is stated that these studies are not sufficient in terms of evidence-based medicine.^12^,^13^

Our primary aim in this study was to determine the performance areas that patients have difficulty in daily life in our chronic stroke patient population. Secondly, it was aimed to investigate the effect of individualized TOT determined in line with the needs of the patients on activities of daily living (ADL) and participation.

## METHODS

The patients who are being treated in Kocaeli University Hospital, Department of Physical Medicine and Rehabilitation (between October 2018 and February 2019) are medically stable, have adequate communication skills, have preserved cognitive functions, do not have severe pain in the UE to affect the treatment, have a spasticity value of two and less than two according to the Modified Ashwort Scale (MAS), have independent sitting balance and over the age of 18 were included in the study. All patients were informed about the study before participating in the study. An informed consent form was signed by each person who agreed to participate in the study. Demographic information of the patients, including age, gender, dominant hand, plegic side, and time after stroke, were recorded. The cognitive levels of the patients were determined by Mini Mental State Exam (MMSE). Accordingly, patients with a test score of 24 and above were included in the study.

### Ethical Approval:

The study was approved by the Non-Interventional Clinical Research Ethics Committee of our hospital with the project number KU GOKAEK 2018/251.

The performance areas in which all patients had limitations before treatment were determined by the Canadian Occupational Performance Measurement (COPM)^14^ independence levels were evaluated with the Modified Frenchay Activity Index (mFAI)^15^ and Barthel Index for Activities of Daily Living (BI).^16^ All assessments were repeated after three weeks of treatment. All assessments were made by an expert with no role in the study.

### Treatment:

All patients were included in the OT program for three weeks, five days a week. Each of the 28 patients was given three ADLs, which were important to them and had difficulty performing, along with various motor skill exercises that would make it easier to perform the activity. Each of the activities within the scope of the prepared treatment plan was designed individually for the patient. Training for all activities (eating using a fork/spoon/knife, fastening buttons, opening and closing zippers, tying shoelaces, vacuuming, washing dishes, ironing, dusting, carrying heavy objects, knitting, typing, using a keyboard, washing hands and face, collecting hair, walking, going up and down stairs, getting up-sitting from a chair and leaning forward) except for the “bathing” activity, was carried out using real objects in the clinical setting. All the movements needed for the bathing activity were disassembled and trained on the patient using the imitation technique.

### Data Analysis:

Statistical analysis was done with the IBM SPSS 20.0 (IBM Corp, Armonk, NY, USA) package program. Normal distribution was evaluated with the Shapiro-Wilk Test. Normally distributed numerical variables were given as mean±standard deviation, non-normally distributed numerical variables were given as median (25th-75th percentile), and categorical variables were given as frequency (percentage). Differences between dependent samples were analyzed by paired t-test and Wilcoxon signed-rank test. Relationships between numerical variables were determined by Spearman and Pearson correlation analysis. Testing two-sided hypotheses was also considered sufficient for p<0.05 statistical significance.

## RESULTS

Among forty-five stroke patients, 28 patients who met the study criteria were included. It included 12 females and 16 males, diagnosed with stroke were included in the study. The mean MMSE of the patients participating in the study was 27.6±1.7. The mean age of the patients was calculated as 47.6±14.8 years. The patients’ demographic information regarding age, gender, dominant hand, hemiplegic side and time after stroke are given in Table-I.

**Table-I:** Demographic information.

Age (years)	47.6±14.8	
Gender	12 women (42.9%)	16 men (57.1%)
Dominant Hand	25 right (89.3%)	3 left (10.7%)
Hemiplegic Side	15 right (53.6%)	13 left (46.4%)
Time after stroke (months)	44.92±11.35	

The evaluations of COPM, mFAI, and BI of stroke patients before and after treatment are given in Table-II. While the average of the COPM performance of the patients before the treatment was 2.91±1.26, the average of the COPM performance after the treatment was 6.38±1.41. A statistically significant increase was found in the COPM performance value compared to the pre-treatment value (p<0.001). The mean COPM satisfaction of the patients before the treatment was 2.43±1.15. After the treatment, the mean satisfaction with the COPM was evaluated as 6.77±1.26, and it was found to be statistically significantly higher than before the treatment (p<0.001). While the mean mFAI was 20.75±4.76 before treatment, this value was 25.92±5.59 after treatment.

**Table-II:** Average values of COPM- BI- mFAI.

	Before Treatment (average ±SD)	After Treatment (average ±SD)	p
COPM–Performance	2.91±1.26	6.38±1.41	p<0.001
COPM- Satisfaction	2.43±1.15	6.77±1.26	p<0.001
BI	84.0±10.0	93.0±9.06	p<0.001
mFAI	20.75±4.76	25.92±5.59	p<0.001

A statistically significant difference was found between pre and post-treatment mFAI values (p<0.001). While the mean BI was 84.0±10.0 before treatment, this value was measured as 93.0±9.06 after treatment, and it was found to be statistically significant after treatment (p<0.001). Percentage change amounts of evaluation parameters were also calculated. According to this, while the average change in the percentage change in the COPM performance was 178.52±226.68, the average change in the percentage change in the satisfaction in the COPM was determined as 258.06±248.61. BI percent change mean: 12.25±8.17, while mFAI percent change mean: 25.32±6.48 (Table-III). In addition, the number and percentage rates of the activities that stroke patients most want to be treated as a result of the COPM evaluations are given in Table-IV.

**Table-III:** Average percentages of change in COPM- BI- mFAI.

	Percentage of Change (average ±SD)
COPM–Performance	178.52±226.68
COPM- Satisfaction	258.06±248.61
BI	12.25±8.17
mFAI	25.32±6.48

**Table-IV:** Activities most preferred by stroke patients.

18 Patients	Dressing Activities: Buttoning Up, Zippering, Tieing Shoelaces	64%
14 Patients	Small Daily Jobs: Cleaning the House, Washing the Dishes, Ironing, Dusting, Carrying Heavy Objects, Knitting, Typing, Using the Keyboard	50%
13 Patients	Hygiene: Washing Hands and Faces, Taking a Bath, To Brush Hair	46%
12 Patients	Using Fork/Spoon/Knife in Eating Activity	42%
11 Patients	Transfer Activities: Walking, Stair Climbing, Sitting from a Chair, Leaning Forward	39%

## DISCUSSION

The purpose of this study was to determine the ADL areas where patients have difficulty in our chronic stroke patient population and to determine the functional contribution of TOT to these activities. In this context, COPM, mFAI and BI tests were used to evaluate the functional status of the patients participating in the study in ADL before and three weeks after the treatment. Within the scope of TOT, all patients were allowed to work on the participation and ADL areas they had difficulty with for three weeks. With this study, first of all, the performance areas where the patients had difficulty in daily life were determined. Second, a statistically significant improvement in all assessment parameters was achieved with three weeks of individualized TOT.

The level of independence in ADL in stroke patients is frequently evaluated with the standardized BI and mFAI. In addition to the Barthel and mFAI scales in our study, in order to determine the effects of task-oriented, individualized treatment, COPM, an individualized assessment method, has been added.

In this study, according to the COPM results, an increase of 3.6 and 4.4 points was observed in the activity performance and satisfaction scores of the patients after the treatment, respectively. It is stated that an improvement of two points or more in COPM provides a moderate-high level of clinical improvement and reflects a serious performance change compared to stroke patients and their families.^17^ In a randomized controlled study conducted by K.A Almhdawi et al. with 20 patients, the effectiveness of TOT was investigated, and they found that there were statistically significant improvements in COPM parameters in the TOT group compared to the control group.^18^ The same author stated that the treatment with the TOT study, the homework given, the intensity of the training, the meaningfulness of the activities and their transferability to daily life activities may help reduce the effects of the “learned non-use phenomenon”^19^ seen in stroke patients. Another randomized controlled study investigating the effectiveness of TOT was conducted by Seonja Park and Bae Seonyoung in Korea. Researchers found a significant improvement in COPM parameters in the TOT group compared to the control group.^20^

According to COPM, most patients had limitations in activities requiring fine dexterity and using both hands in our study. The “eating” activity was ranked 4^th^ in the list of preferred activities since there is no obligation to use two hands in the eating activity, and the patient can do this activity with the healthy side UE. On the other hand, the presence of the lower extremity on the healthy side and the use of large muscle groups in activities such as walking, climbing stairs or getting up from a chair suggests that patients have less difficulty in such performance areas. When studies investigating the effectiveness of TOT were examined in the literature, no study was found that categorizes the performance areas that stroke patients have difficulty performing. Jin-Uk Choi and Soon-hee Kang with 20 stroke patients in 2015, COPM was used to select the tasks to be used in treatment. The same study stated that patients who received TOT training showed positive improvements in all evaluation parameters, including COPM, compared to the control group.^21^

A statistically significant improvement was observed in BI scores in our study. In Fernandes et al. TOT effectiveness research, it was observed that the BI scores of the study group increased by 39 points compared to the beginning. It was stated that statistically significant improvements were seen in BI scores after 12 weeks of treatment and that TOT-based therapies contributed to the acquisition of functional independence^22^. In the randomized controlled study conducted by Alsubiheen, A. M. et al. in 2022, one group received TOT training while the other group received conventional OT, and although there was an improvement in the scales measuring manual dexterity and BI parameters in both groups, a significant improvement was achieved in favor of the TOT group.^23^

Significant improvements were achieved in the mFAI scale, one of the evaluation scales of our study, compared to the pre-treatment period. When the current literature was examined, no study was found that used mFAI as an evaluation scale in studies investigating the effectiveness of TOT. We think that this study we have conducted is the first on this subject.

It has been reported that 80% of stroke patients have problems with hand functions due to hemiplegia, making patients dependent on ADL. Functional disorders in the UE are one of the first obstacles to gaining competence in the management of ADL. Considering the studies examining UE dysfunctions in stroke, it has been reported that the level of functionality is highly correlated with UE and hand motor deficiencies.^24^,^25^

In recent years, a new treatment method called TOT has begun to be used in stroke rehabilitation in addition to conventional treatments. TOT, which is a treatment option used to increase the effectiveness of UE in ADL, is a clinical therapeutic approach based on rehabilitation science, motor learning is based on the principles of motor control and neuroplasticity.^26^

It is stated that TOT is more effective in functional recovery compared to general exercise models in stroke patients.^27^ TOT has been reported to be effective in improving the functional motor skills necessary to perform ADLs, according to several recent studies conducted on stroke patients. Researchers note that TOT consists of a variety of functional activities that provide a more effective treatment approach, allowing patients to perform ADLs.^28^-^30^

No study conducted in our country on the effectiveness of TOT in this disease population has been found in the literature. However, in the general literature review, it is seen that there are studies similar to ours.^11^,^18^,^23^ All these studies showed that the task-oriented and individualized treatment approach contributed significantly to the functional development of UE, and improvements were achieved in the COPM and quality of life scales of stroke individuals.

### Limitations:

It include the inability to control the patients’ daily life outside of therapy sessions and the lack of a follow-up test to assess the long-term effect. Another limitation of the study is the absence of a control group to evaluate TOT. In addition, due to the study’s small sample size, it is difficult to generalize the findings to other stroke patients. Randomized controlled studies with larger numbers of patients are needed.

## CONCLUSIONS

Based on this study, we believe that adding TOT to the classical treatment program will contribute to the treatment to improve ADL in the rehabilitation programs of stroke patients.

### Authors’ Contributions:

**CC** and **IS:** Designed this study, prepared this manuscript, are responsible and accountable for the accuracy and integrity of the work.

**CC** and **EO:** Collected and analyzed clinical data.

**SB** and **CC:** Data analysis, significantly revised this manuscript, analysis, and interpretation of data and draft the manuscript
